# Alvarez varifocal X-ray lens

**DOI:** 10.1038/s41467-023-40347-1

**Published:** 2023-07-31

**Authors:** Vishal Dhamgaye, David Laundy, Hossein Khosroabadi, Thomas Moxham, Sara Baldock, Oliver Fox, Kawal Sawhney

**Affiliations:** 1 Diamond Light Source, Harwell Science and Innovation Campus, Didcot, Oxon OX11 0DE UK; 2 Synchrotron Utilisation Section, Raja Ramanna Centre for Advanced Technology, Indore, India; 3 Department of Engineering Science, University of Oxford, Parks Road, Oxford, Oxon OX1 3PJ UK; 4 Department of Chemistry, Lancaster University, Lancaster, LA1 4YB UK

**Keywords:** X-rays, Micro-optics

## Abstract

Visible light optical elements such as lenses and mirrors have counterparts for X-rays. In the visible regime, a variable focusing power can be achieved by an Alvarez lens which consists of a pair of inline planar refractors with a cubic thickness profile. When the two refractors are laterally displaced in opposite directions, the parabolic component of the wavefront is changed resulting in a longitudinal displacement of the focus. This paper reports an implementation of this concept for X-rays using two planar microfabricated refractive elements. The Alvarez X-ray lens can vary the focal distance of an elliptical X-ray mirror or a planar compound X-ray lens over several millimetres. The study presents the first demonstration of an Alvarez X-ray lens which adaptively corrects defocus and astigmatism aberrations of X-ray optics. In addition, the Alvarez X-ray lens eliminates coma aberration in an elliptical mirror, to the lowest order, when combining the lens with an adjustment of the pitch angle of the mirror.

## Abstract (web summary)

Development of synchroton facilities increases the demand of optics for focusing X-ray beam to achieve diffraction-limited focusing onto samples. Here, the authors demonstrated an X-ray optical element based on visible light Alvarez varifocal lens providing precise performance focusing elements.

## Introduction

Many major synchrotron facilities have already done or are planning to do lattice upgrades to reduce the source emittance and increase the X-ray brightness and coherence^1^ and in parallel, advances in the manufacturing of optical elements allow the high X-ray coherence to be exploited to give diffraction-limited focusing of the X-rays on to samples^2,3^. High stability and precise alignment of the optics are required^4^ and in future, correction of the X-ray wavefront^5–7^ may be employed. Aberrations in the X-ray focusing system may be caused by optics misalignment or fabrication errors. In the case of a Kirkpatrick–Baez (KB) mirror system^8^, which consists of two elliptical mirrors, independently focusing in horizontal and vertical directions, astigmatism can be caused by a displacement of one mirror along the optical axis or by an offset of the mirror pitch angle. In the case of compound X-ray refractive lens (CRL), astigmatism may be introduced during the fabrication process causing a distortion of the lens profile, mispositioning of individual lenses in the lens stack, or angular misalignment of the overall stack of lenses^9^. Defocus aberration occurs when the sample is displaced from the focal plane. In practice, the sample may not be moveable if it is constrained inside a sample environmental chamber. In addition, for CRLs, a change in the X-ray energy results in a change to the lens focusing power, which moves the focal plane along the *z* direction. To compensate for these effects, it would be advantageous to have a simple method to independently adjust the longitudinal position of the horizontal focus and the vertical focus of beamline optics.

Control over the focal plane position can be done by lens-based transfocators which work by translating individual lenses into a CRL lens stack to change the focal length in discrete steps^10–12^ and ‘zoom’ optics using a combination of adaptable mirrors^13,14^_._ Focal length variation of an elliptical mirror is also possible by changing the X-ray incidence angle; however, this introduces coma aberration as the X-ray source is then no longer positioned at a geometrical focus point of the mirror ellipse. Here, we present an implementation of a simple adaptive X-ray optical element based on the visible light Alvarez varifocal lens^15,16^ that can change the focusing of X-rays in one direction. In contrast to visible light, for X-rays, the refractive index is close to unity and the Alvarez X-ray lens (AXL) provides only a weak perturbation to the wavefront. Thus, AXL provides a precise varifocal performance of X-ray focusing elements such as an X-ray mirror or an X-ray lens. Using a combination of AXL shearing and mirror pitch offset angle, we demonstrate the elimination of the first-order coma aberration in the X-ray mirror.

## Results

**Fig. 1:**
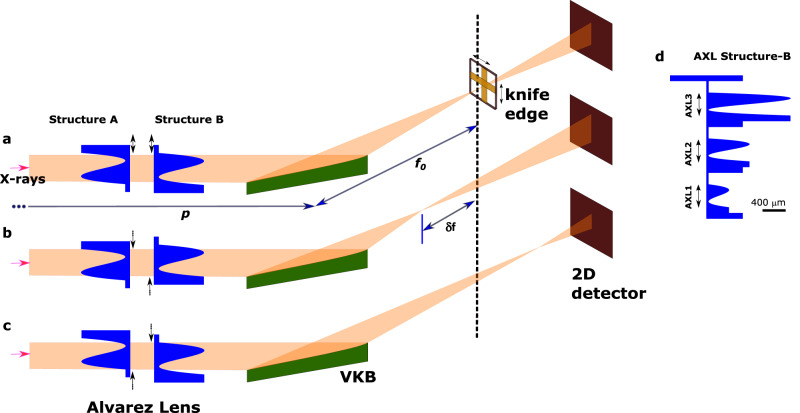
Schematic experimental setup for AXL characterisation with the focusing mirror (VKB). For zero translation between the two structures of AXL (**a**), the outgoing wavefront of VKB remains unaffected. For opposing translations, a positive or negative parabolic perturbation of the wavefront results (**b**, **c**). Inset (**d**) Profile of one of the two fabricated AXL structures viewed from the side. Each of the three AXL refractors (AXL1, AXL2, AXL3) can be moved into the X-ray beam by a vertical translation.

The AXL consists of two refractors in line with the X-ray beam. Using cartesian coordinates $\left(x,\;y,\;z\right)$, a planar X-ray wavefront incident on the AXL propagates along direction, $z$, and the optical element focuses the X-rays in the $y$ (vertical) direction (see Fig. 1). The two AXL refractors can each be independently translated in the $y$ direction and their X-ray path lengths along $z$ are:

1 $${t}_{1}\left({{{{{\rm{y}}}}}}\right)={+A}_{3}{\left(y+\triangle \right)}^{3}+{A}_{1}\left(y+\triangle \right)+{A}_{0}$$

2 $${t}_{2}({{{{{\rm{y}}}}}})=-{A}_{3}{(y-\triangle )}^{3}-{A}_{1}\left(y-\triangle \right)+{A}_{0}$$

where ${A}_{0}$, ${A}_{1}$ and ${A}_{3}$ are constants and $\Delta$ is a variable differential offset (shearing) of the two refractors along the $y$ direction. In our AXL design, each refractor structure has a flat surface on one side and a cubic surface on the other side as defined by above two equations. The coefficients of the cubic polynomial for AXL1 were ${A}_{0}$ = 0.284 mm, ${A}_{1}$ = −4.25 and ${A}_{3}$ = 141.5 mm^−2^ and the parameters for AXL2 and AXL3 are two times and four times bigger. Both of the structures had a support along the *z* direction of thickness ${t}_{0}=$50 µm and side fiducials for their alignment in the X-ray beam.

The combined X-ray path length $t\left(y\right)={t}_{1}\left(y\right)+{t}_{2}\left(y\right)$ is (from Eqs. (1) and (2)):

3 $$t\left({{{{{\rm{y}}}}}}\right)=6{A}_{3}\triangle {y}^{2}+2{A}_{3}{\Delta }^{3}+2{A}_{1}\Delta+2{A}_{0}+2{t}_{0}$$

Ignoring the constant terms, this gives rise to a parabolic perturbation of the X-ray wavefront:

4 $$w\left(y\right)=6\delta \left(E\right){A}_{3}\triangle {y}^{2}$$

where the X-ray refractive index of the medium at X-ray energy $E$ is conventionally written as $1-\delta \left(E\right)$. The dependence of Eq. (4) on ${y}^{2}$ shows that by varying the shearing ($\Delta$), the AXL can apply a variable parabolic perturbation to the X-ray wavefront. The refractive index real-part decrement $\delta \left(E\right)$ for the fabricated AXL is of order ${10}^{-6}$ at X-ray energies and, therefore, the AXL acts as a weak planar lens with focal length ${f}_{A}$ given by:

5 $$1/{f}_{A}=12\Delta {{{{{\rm{\delta }}}}}}\left(E\right){A}_{3}$$

A combination of two AXLs, orientated at ${90}^{\circ }$ about the optical axis - correcting the wavefront in horizontal and vertical directions, would allow simultaneous compensation of both astigmatism and defocus aberrations. As the AXL provides a weak correction to the wavefront, the focal length of the AXL, ${f}_{A}$, is large compared to the separation between the AXL and the optical element and is also large compared to the focal length of the optical element. If the focal length of the optical element is ${f}_{0}$, then the thin lens formula gives the modified focal length**:**

$$\frac{1}{f}=\frac{1}{{f}_{0}}+\frac{1}{{f}_{A}}.$$

In the case that the separation (*d*) between the AXL and the optical element is significant, then the modified focal length (with respect to the optical element) is given by the back focal length^17^:

6 $$\frac{1}{f}=\frac{1}{{f}_{0}}+\frac{1}{{f}_{A}-d}$$

Further, defining ${f}_{A}^{{\prime} }={f}_{A}-d$ and if ${f}_{A}\;\gg \;{f}_{0}$ this can be simplified to give:

7 $$f=\,{f}_{0}/(1+{f}_{0}/{f}_{A}^{{\prime} })\, \sim \,{f}_{0}\left(1-{f}_{0}/{f}_{A}^{{\prime} }\right)$$

and therefore, the change in the focal length, $\delta f$, caused by introducing the AXL is:

8 $$\delta f={f}_{0}-f \sim -{f}_{0}^{2}/{f}_{A}^{{\prime} }$$

As can be seen, the focal length range scales as the square of the focal length of the optical element and shearing for an AXL. Three AXLs with different parameters (${A}_{0},{A}_{1},{A}_{3}$) were fabricated—the details are described in the Methods section. The AXL was placed upstream of the X-ray focusing element and the schematic experimental setup is shown in Fig. 1. The AXLs were then tested using monochromatic X-rays with two different X-ray focusing optical elements—a vertically focusing mirror (VKB)—part of a KB mirror pair^8^ and a vertical focusing compound planar X-ray lens. In addition, the operation of the AXL was simulated using the X-ray ray tracing programme Shadow^18,19^. Measurements were carried out on the Optics Test beamline at Diamond Light Source (B16) where broadband X-rays from the dipole source were monochromatised by a double crystal silicon 111 monochromator to give X-rays with an energy of 15 keV. The optical elements and the AXL were aligned using the knife-edge wavefront measurement method^20^. With this method, the wavefront error – the difference between the actual wavefront and a perfect wavefront at the optical element is measured. A displacement of the knife edge along *the z* direction, therefore, results in a parabolic contribution to the wavefront and higher order polynomial components to the wavefront are caused by figure errors (for focusing mirrors), profile errors (for lenses) or optics misalignment such as an incorrect pitch angle for a mirror.

### Calibration of the AXL

**Fig. 2:**
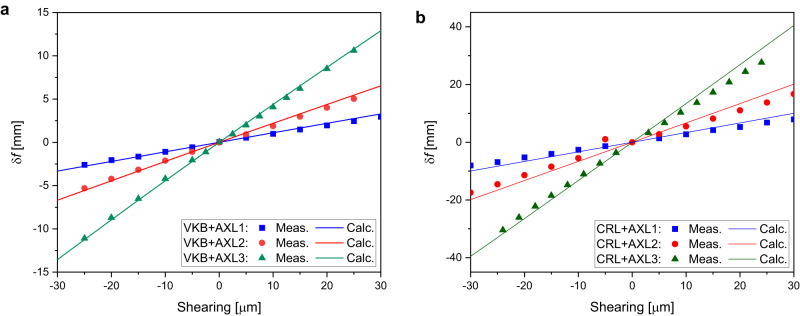
AXL varifocal calibration curve for VKB and CRL. Measured change in focus position of (**a**) VKB mirror and (**b**) CRL as shearing varied in AXL1, AXL2 and AXL3 is illustrated. The solid line represents the calculated values obtained using Eq. (8).

The AXL was initially set up with shearing $\Delta=0$ (Fig. 1a) and the focal plane of the optical element was then located. With a shearing applied, a concave (Fig. 1b) or convex (Fig. 1c) parabolic wavefront perturbation results. The measured change in focus position for X-rays of energy 15 keV as the AXL shearing ($\Delta$) changed is shown for the VKB elliptical mirror in Fig. 2a and for the planar CRL in Fig. 2b. The calculated solid lines are the result of the application of Eq. (8). A change in the mirror focal length of 102, 206 and 428 μm per 1 μm shearing in AXL1, AXL2 and AXL3, respectively, was obtained. The corresponding change in the CRL focal length was 270, 570 and 1200 μm per 1 μm shearing in AXL1, AXL2 and AXL3, respectively.

### Varifocal performance of an elliptical mirror with an AXL

**Fig. 3:**
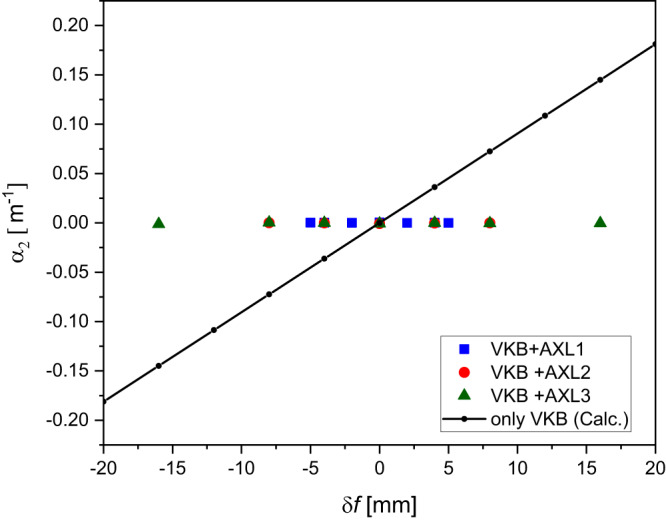
VKB defocus coefficient with and without AXL. The dots show the measured change in defocus coefficients of the outgoing wavefield of VKB with Alvarez X-ray lenses for different knife-edge positions ±16 mm around VKB’s focal length. The role of the AXL is apparent in keeping defocus term of the elliptical mirror constant which would otherwise vary linearly along the *z* direction.

The AXL produces a variable focal length when combined with a focusing optical element such as an elliptical mirror or lenses. Figure 3 shows the zero parabolic component of the wavefront due to the introduction of the AXL which otherwise changes linearly in the longitudinal focal plane ±20 mm. The obtained range of focal plane movement is larger than VKB’s depth of focus, 250 µm at 15 keV. The resolution of focal plane movement is ~<1 μm, which is very small compared to the depth-of-focus. AXL shearing was carried by a nano-positioner stage which shows no sign of instability of involved optics and the time required for a 1 mm focal plane change is approximately 5 ms.

#### Coma compensation for an elliptical mirror

**Fig. 4:**
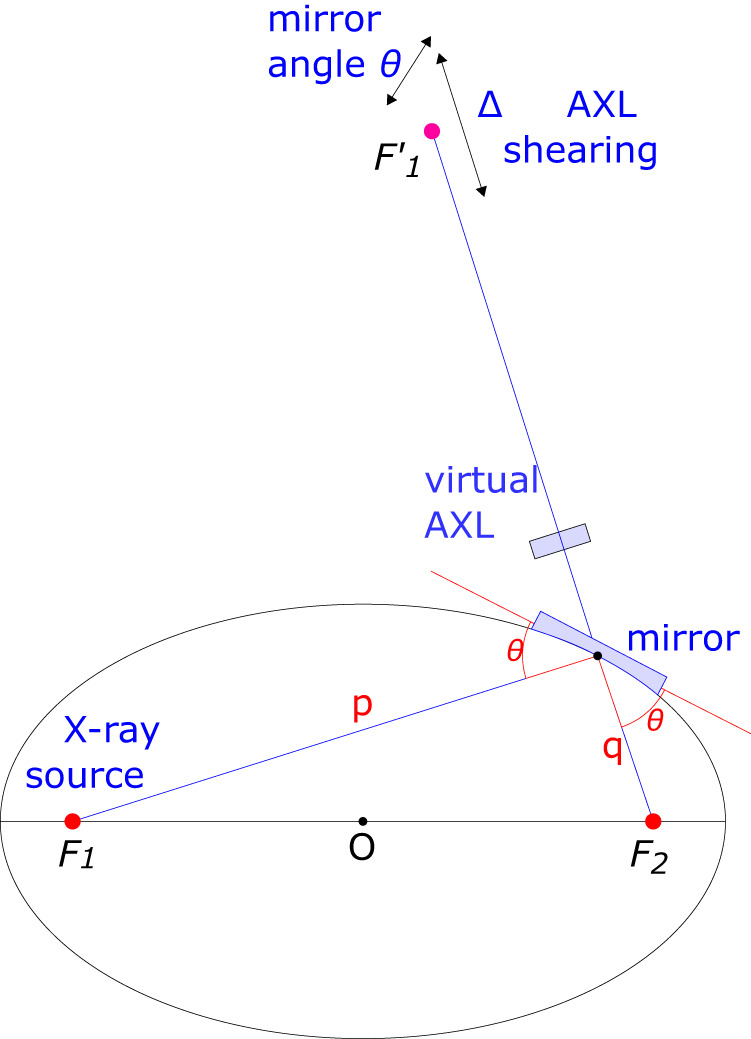
Focusing by an elliptical mirror with pitch angle *θ*. Rays from the source at ${F}_{1}$ are reflected from the mirror and focused to the point ${F}_{2}$, giving rise to a virtual source ${F}_{1}^{{\prime} }$. As shown, a change to the mirror pitch angle results in an oblique translation of the virtual source and a change to the AXL shearing gives rise to a longitudinal translation of the virtual source along the optical axis.

For a highly demagnifying elliptical mirror such as the VKB mirror studied here, we investigated whether the AXL can be used to minimise coma aberration of the focus when the VKB is misaligned in pitch angle. Positioned upstream of a focusing optical element, the AXL converts the real X-ray source into a virtual source that is displaced in the longitudinal direction. With a perfect elliptical mirror (see Fig. 4), completely coma free focusing only occurs when the source is positioned exactly at the ellipse point ${F}_{1}$ and the beam focus is then at ellipse point ${F}_{2}$. The AXL moves the virtual source away from point ${F}_{1}{\prime}$ and therefore introduces coma onto the focus.

The X-ray wavefront is a useful concept for simulating the effect of aberrations in optical elements. For an ideally focused wavefront, the wavefront curvature is constant, and the field propagates along the wavefront’s normal direction to the focal plane where the field amplitudes are all in phase and interfere constructively to give a diffraction-limited focus. The wavefront error is the deviation of the wavefront from this ideal shape. Aberrations show up as a distortion of the wavefront so that the fields are not completely in phase at the focal plane.

For an elliptical mirror, a virtual source can be constructed by propagating the wavefront back to a source plane, as shown in Fig. 4. A change in the mirror pitch angle $\theta$ moves the virtual source along a combination of the transverse direction (perpendicular to the optical axis) and the longitudinal direction (along the optical axis) caused by the dependence of the focal length on $\theta$. The shearing of the AXL adds a parabolic component to the wavefront which moves the virtual source along the longitudinal direction.

As the AXL shearing and the mirror pitch angle move the virtual source in non-collinear directions, within the 2-dimensional plane ($\Delta,\;{{{{{\rm{\delta }}}}}}\theta$) where $\delta \theta$ is the mirror pitch angle offset, the virtual source can be positioned anywhere in that plane. The wavefront error after the mirror can be expressed as a power series:

9 $$w\left(y\right)={\alpha }_{0}\left(\Delta,\;\delta {{{{{\rm{\theta }}}}}}\right)+{\alpha }_{1}\left(\Delta,\;\delta {{{{{\rm{\theta }}}}}}\right)y+{\alpha }_{2}\left(\Delta,\;\delta {{{{{\rm{\theta }}}}}}\right){y}^{2}+{\alpha }_{3}\left(\Delta,\;\delta {{{{{\rm{\theta }}}}}}\right){y}^{3}+\ldots$$

where every coefficient ${\alpha }_{i}\left(\Delta,\;\delta \theta \right)$ is zero when both $\Delta$ and $\delta \theta$ are zero. The ${\alpha }_{0}$ term which causes a constant phase change of the wavefront and the ${\alpha }_{1}$ term which causes a linear variation of phase with position, equivalent to a tilt of the wavefront can be ignored. The coma aberration can therefore be eliminated up to the third order term if there exists a locus of points in the (Δ, $\delta \theta$) plane on which the ${\alpha }_{3}$ term is zero, effectively counteracting an offset in the mirror pitch angle by AXL shearing.

**Fig. 5:**
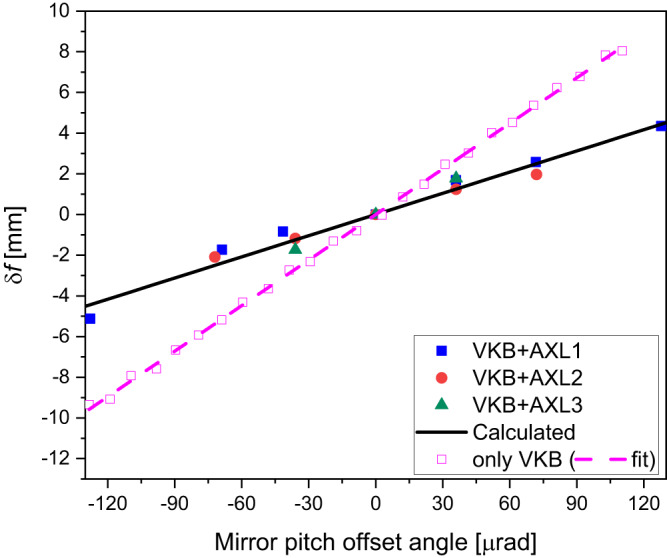
Coma free VKB focus variation with VKB pitch angle offset (*δθ*) and AXL shearing (Δ). The open squares show *δf* obtained by pitch angle change only and other dots show *δf* variation with the cubic term reduced to zero by the three AXLs—AXL1, AXL2 and AXL3.

**Fig. 6:**
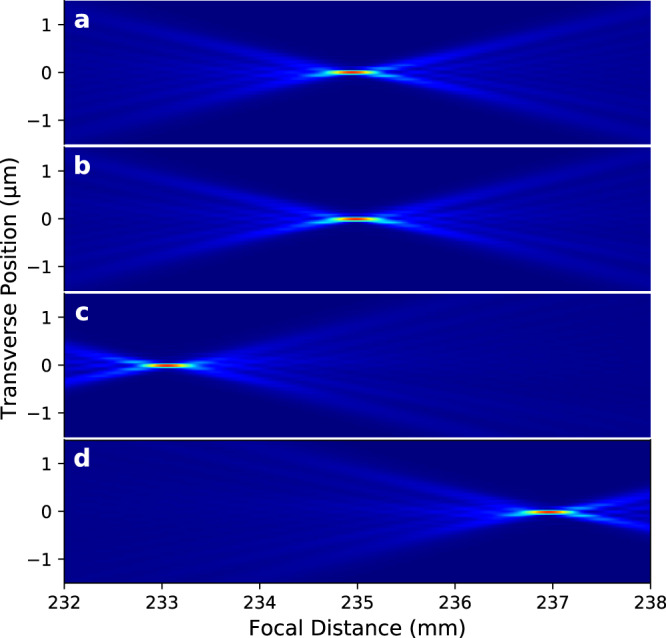
VKB beam caustics. The beam Intensity distribution after numerical propagation of the measured complex field to the intended focal positions of (**a**) VKB at 235 mm (**b**) VKB with aligned AXL2 at 235 mm, and when defocus and coma aberrations are minimised (**c**) and (**d**) VKB with AXL2 at 235 mm $\sim \mp$2 mm, after shearing of AXL structures $\triangle$ = $\mp$15 µm and $\triangle$ = $\pm$15 µm, and VKB pitch angle offset $\delta \theta$ = $\mp$72 µrad, respectively.

**Fig. 7:**
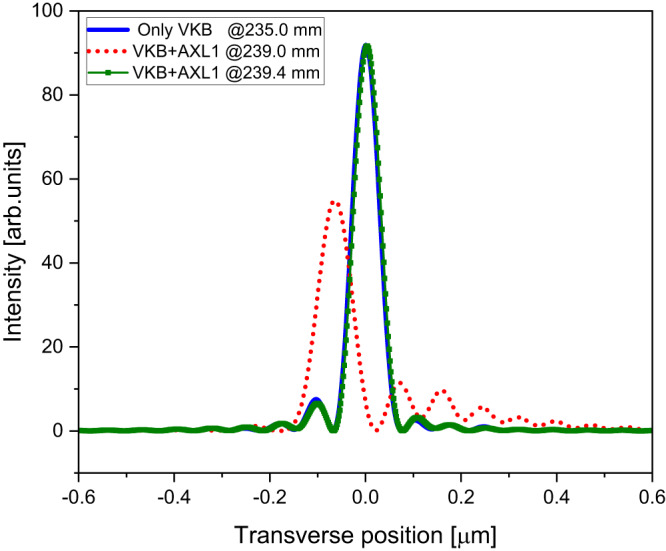
Focused beam profiles of the VKB shifted focal planes. The solid line shows the intensity profile at the VKB focal plane (235 mm from the mirror centre) with the correct incident angle for the mirror (3 mrad) and with the AXL shearing at zero. The dashed line shows the focus profile of VKB focal plane displaced by 4.0 mm after a shearing of AXL1 ($\triangle$ = $\pm$39.7 µm). The solid line with a square dot shows the focus profile of 4.4 mm displaced VKB focal plane by adjusting the mirror pitch offset angle ($\delta \theta=$127.8 µrad) and then adjusting the shearing ($\triangle$ = $\pm$45.4 µm) to remove the coma aberration.

The correction was implemented by firstly offsetting the mirror pitch angle ($\delta \theta$) and at a fixed *z* position of the knife edge, measuring the wavefront error $w\left(y\right)$ for a few values of the AXL shearing ($\Delta$). For each AXL shearing, the polynomial coefficients ${\alpha }_{i}\left(\Delta,{{{{{\rm{\delta }}}}}}{{{{{\rm{\theta }}}}}}\right)$ were obtained by linear regression of the measured wavefront error $w\left(y\right)$. The ${\alpha }_{3}\left(\Delta,{{{{{\rm{\delta }}}}}}{{{{{\rm{\theta }}}}}}\right)$ coefficient was then extrapolated linearly to zero to give the required AXL shearing $\Delta$ for which ${\alpha }_{3}\left(\Delta,\delta \theta \right)=0$. At this value of AXL shearing, the ${\alpha }_{2}$ term is the targeted focal position change, which can be measured by varying the knife edge *z* position to reduce the second order term to zero. After these adjustments, the wavefront error consists of only 4th order terms and above. This result was verified by raytracing which showed that for fixed shearing of the AXL, there is a single value of the mirror pitch angle at which the 3rd order component of the wavefront becomes zero and that the focal shift of this combination is smaller than can be obtained by a change of the AXL shearing only or by a change in mirror pitch angle only. In our measurement, control of the mirror pitch angle could be unreliable and, therefore, we fixed the mirror pitch angle while the AXL shearing was varied to remove the cubic component. Figure 5 shows the variation of focal plane position with and without (only VKB) AXL and the cubic term compensation for a change in the mirror pitch angles. The measured wavefronts were used to calculate the complex field, which was then propagated to positions around the focal plane using the Fresnel–Kirchhoff equation^21^. Figure 6 shows beam caustics of the VKB at the ideal and shifted focal planes with AXL2 used to eliminate the lowest order coma aberration. Figure 7 shows resulting focus profiles obtained from the beam caustics (please see Supplementary Fig. 1) of the VKB at its ideal focal plane, at the shifted VKB focal plane 4 mm downstream by AXL1 shearing which corrects only defocus and at the shifted VKB focal plane by the combination of VKB pitch angle and AXL1 shearing that corrects defocus and first-order coma. This shows that by compensating the 3rd order contribution to the wavefront with the AXL, the focal plane has been moved while still providing a diffraction-limited focus profile close to the result obtained by the mirror alone. AXL has a potential to use in the existing beamlines along with high demagnification optics to vary their focal lengths and retain their diffraction-limited focus profile.

### Varifocal performance of the CRL with an AXL

**Fig. 8:**
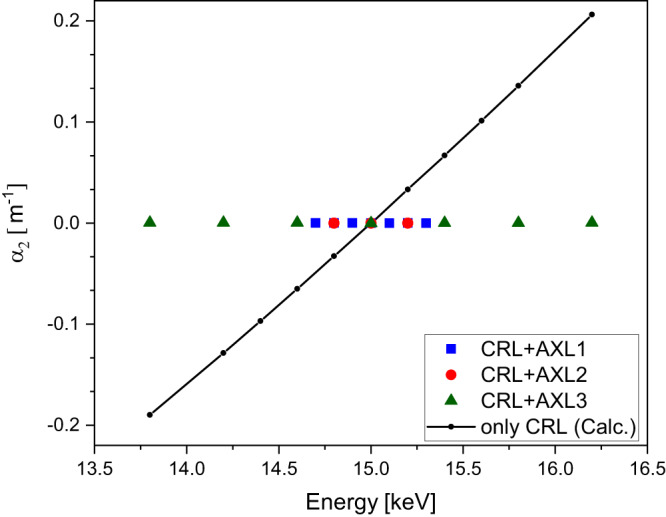
CRL defocus coefficient with and without AXL. The dots show the measured change in defocus coefficient of the CRL wavefront in the energy range ±1.2 keV around 15 keV monochromatic beam and calculated coefficients without AXL (solid line with circle). The location of the focus place was found approximately constant (variation of −40 to 90 µm) for 13.8 keV to 16.2 keV, which otherwise shifted in the same energy range from −64 mm to 69 mm.

With CRLs, the wavefront error is often symmetrical about the optical axis so the cubic term is not significant. The experimental setup was similar to the elliptical mirror setup with the AXL positioned upstream of the planar vertically focusing CRL. For X-rays, the refractive index is energy dependent and therefore a change in X-ray energy results in a longitudinal shift of the CRL focal plane. This can be compensated by an AXL using the shearing to achieve a constant focal plane position. The calibration curves of the three AXLs obtained at 15 keV and AXL shearing required at different energies are illustrated in Fig. 2b and Supplementary Fig. 2, respectively. Figure 8 shows the relative change in parabolic coefficients of measured wavefronts as a function of X-ray energies for AXL1 to AXL3 with the CRL. The experimental results show that the focal distance of CRL remains constant for a wider energy range which also agrees well with the corresponding calculated values. For the CRL studied, AXL3 could be used to control the focal length at 15 keV over an energy range of ±1.2 keV.

## Discussion

We have successfully developed and demonstrated the Alvarez lens operating in the X-ray region for adaptive control of the focal length of an elliptical mirror and a compound X-ray lens. By using two orthogonally orientated AXLs, astigmatism and defocus could be simultaneously corrected. We have also shown that coma in elliptical mirrors can be compensated to first order by combining the AXL shearing with a mirror pitch angle change. This allows the focus distance to be changed over a large range in order to correct mispositioning of the two mirrors in a KB pair or to adjust to different sample positions. The AXL is compact, is mounted in-line with the X-ray beam and can be positioned upstream of the focusing optical element. An AXL could therefore be inserted into an existing optical layout and easily translated out of the beam path when not required. As the optical axis is not deviated, the alignment is easy and with better goniometry, AXL shearing could be changed within a fraction of a second.

For CRLs, an AXL can translate the X-ray focus position for alignment of the focus to samples and can compensate for the focus position change that occurs when the X-ray energy is changed. AXLs could be used in a range of X-ray experiments at modern synchrotron radiation sources such as nanoprobe experiments, micro-crystallography and imaging. In addition, an AXL could be used to correct for astigmatic wavefront errors such as those caused by thermal distortion on upstream optical elements.

## Methods

### Fabrication

The AXLs are composed of the polymer IP-S, molecular formula C_14_H_18_O_7_ and density 1.2 g/cm^3^. The IP-S X-ray optical constants were calculated using the programme XOP^22^. A 3D layout of the AXL for fabrication by 3D printing was prepared using a CAD package. The design data of Structure-B for all AXLs in three- and two-dimensions are illustrated in Supplementary Fig. 3. The structure-A and structure-B of each AXL were printed in photoresist on two separate glass substrates of cross section 25 × 25 mm^2^. A small amount of IP-S photoresist (Nanoscribe GmbH) was dropped on the glass substrate. The commercially available 3D printer (Photonic Professional GT, Nanoscribe GmbH) was used in dip-in-lithography mode at laser wavelength 780 nm for two-photon polymerisation. The photoresist was exposed in the bottom-to-top approach with the laser pulse focused into each voxel by a high-resolution objective (x25 NA 0.8), laser settings were 38 mW power and writing speed was 100,000 µm/s. The 3D CAD structures were formatted into printing commands using Nanoscribe proprietary software (DeScribe 2.5.5) using the following print settings; vertical slice: 0.5 µm, hatch: 0.25 µm, block stitching: 200 × 200 × 298 µm^3^, 15° angle, 2 µm overlap. The photoresist after printing was developed in Propylene glycol methyl ether acetate (PGMEA) for 30 min, rinsed in IPA for 5 min and dried with Nitrogen gas.

The X-ray absorption of the AXL increases from AXL1 to AXL3 due increase in the X-ray path length. The calculated transmissions for AXL1, AXL2 and AXL3 are 90%, 82% and 70% for $\pm$40 μm shearing, respectively, at 15 keV. The radiation resistance of IP-S has been reported elsewhere and shows that material shrinkage occurs with X-ray dose in excess of 15 MGy in 5.5 h when exposed to an undulator beam^23^. The AXL characterisation reported here was carried out on monochromated ($\Delta E/E \sim {10}^{-4}$) bending magnet source with incident photon flux ~2.6 × 10^9^ photons/s/mm^2^ which would require 3600 h to show a similar dose to the undulator dose.

### Optics characterisation setup

Experiments were performed with a monochromatic beam on the B16 Test beamline of the Diamond Light Source^24^. The schematic arrangement of the AXL for varying the VKB focal length is shown in the experimental layout (please see Fig. 1). The KB mirror system consisted of two elliptical mirrors, each fabricated from single crystal silicon with a rhodium coating, oriented orthogonally to each other to provide a focus at a common focal plane. Both elliptical mirrors had a 3 mrad grazing incidence angle and 90 mm active length. The planar CRL consisted of 27 bi-concave parabolic lenses with on axis radius of 25 µm fabricated by deep X-ray lithography out of the polymer SU-8 and laid down on a silicon wafer substrate.

The two AXL structures were installed on two separate motion towers, each with five degrees of freedom and were placed upstream of the KB mirror system rather than downstream where the working distance is smaller. The distance between the two AXLs in the prototype was approximately 63 mm, however, in principle, this distance could be reduced considerably. The distance between the centre of the AXL to VKB was *d* = ~340 mm (359 mm for CRL) limited by the KB mirror vacuum vessel and the AXL mounting platform. A knife-edge scanning in the vertical or horizontal direction was placed in the KB mirrors’ focal plane and the beam intensity variation due to scanning the knife-edge through the beam was recorded using a 6.5 μm pixel area detector (Mini-FDS from Photonic Science). Each AXL and the knife-edge were mounted on nano-positioning linear stages (Attocube) that has resolution <10 nm and angular nano-positioner stages (Attocube) has a resolution <0.001°.

### Wavefront measurement and propagation

X-ray wavefront measurements gives information about path-length error^5,25–27^ and can be used for precise optics alignment^28,29^. Previously, the residual wavefront error of Be CRLs were measured using X-ray ptychography and the knife-edge method and showed good agreement^30^. The knife-edge method was used in the present study to measure wavefronts of optical elements^20^. A microfabricated gold knife-edge was scanned vertically or horizontally near to the focal plane of the focusing optical element and the intensity measured on an X-ray area detector at distance of 0.5 m to 1 m downstream of the focus. The one-dimensional wavefront error profile was extracted from the intensity variation in each detector pixel during the knife-edge scan. The wavefront error was fitted with a 3rd order polynomial. The second order coefficient gives the defocus aberration, and the remaining terms (3rd order and above) are responsible for coma aberration. The measured intensity was numerically propagated using the Fresnel–Kirchhoff equation to investigate a change in the beam caustic and variation in the focal length of the mirror.

### Simulations

Simulations were done using the Shadow raytracing programme^18,19^. The layout and all parameters have been set from the experiment (Fig. 1). No slope errors and fabrication errors have been implemented to keep the ideal focusing condition. The number of rays was 1 million and a point source were used. The wavefront was calculated for a set of mirror pitch angles and knife edge positions along the optical axis using knife-edge imaging method^20^.

### Alignment of focusing optics and AXL

Alignment of the KB mirrors: For the initial alignment of the mirror, the mirror pitch angle was adjusted to give a zero cubic component in the wavefront error. Then, the knife edge position along the optical axis was adjusted to give a zero parabolic component to the wavefront error. The incidence angle was optimised by finding the angle where the wavefront error cubic component is zero and following optimisation of the pitch angle, the focal plane was located as the position where the parabolic component is zero.

Alignment of the CRL: The CRL was mounted on goniometer with 3 translation axes (x, y, z) and two rotations axes about horizontal axis (*x*) and vertical axis (*y*). The beam was projected onto a downstream X-ray detector and the transmitted intensity was maximised. Then a knife edge was scanned near the focal plane allowing the focal plane to be located by minimising the parabolic component of the wavefront.

Alignment of AXL: Following the alignment of the optical element, the AXL was positioned a short distance upstream of the optical element on a goniometer. The two AXL structures were adjusted in position and angle using fiducial structures incorporated in the design which were visible on a downstream X-ray area detector. This allowed the AXL axis to be aligned in angle and in position to the optical axis. Fine alignment of the differential vertical positions of the two structures was then done by measuring the X-ray wavefront after the optical element and finding the structure positions where the AXL had no effect on the wavefront (corresponding to $\Delta=0$ in Eq. (5)). At this position, the AXL produces a uniform phase shift. We also carried out extensive simulation of the effect of the AXL alignment using ray tracing, which showed that misalignments of 0.05 µm in position and 0.007° in angle are acceptable.

## Supplementary information

Supplementary Information

## Data Availability

The processed data that supports the findings of this study are available in the Zenodo public repository and can be accessed at 10.5281/zenodo.8011822.
