# Supplementary material for: Alvarez varifocal X-ray lens
Source: Nat Commun. 2023 Jul 31;14:4582. doi: 10.1038/s41467-023-40347-1 (PMC10390599; doi:10.1038/s41467-023-40347-1)
Supplement: Supplementary file 1 — Supplementary Information [file 41467_2023_40347_MOESM1_ESM.pdf]

Supplementary Information:

Alvarez varifocal X-ray lens

Vishal Dhamgaye[1, 2], David Laundy[1], Hossein Khosroabadi[1], Thomas Moxham[1, 3], Sara Baldock[4], Oliver Fox[1] and Kawal Sawhney[1]

[1]Diamond Light Source, Harwell Science and Innovation Campus, Didcot, Oxon. OX11 0DE, UK

[2]Synchrotron Utilisation Section, Raja Ramanna Centre for Advanced Technology, Indore, India

[3]Department of Engineering Science, University of Oxford, Parks Road, Oxford, Oxon. OX1 3PJ, UK

[4]Department of Chemistry, Lancaster University, Lancaster, LA1 4YB, UK

In this supplementary material, we provide additional figures; namely, the vkb beam caustic, AXL calibration curve for different energies, and design data for AXL structure-B.

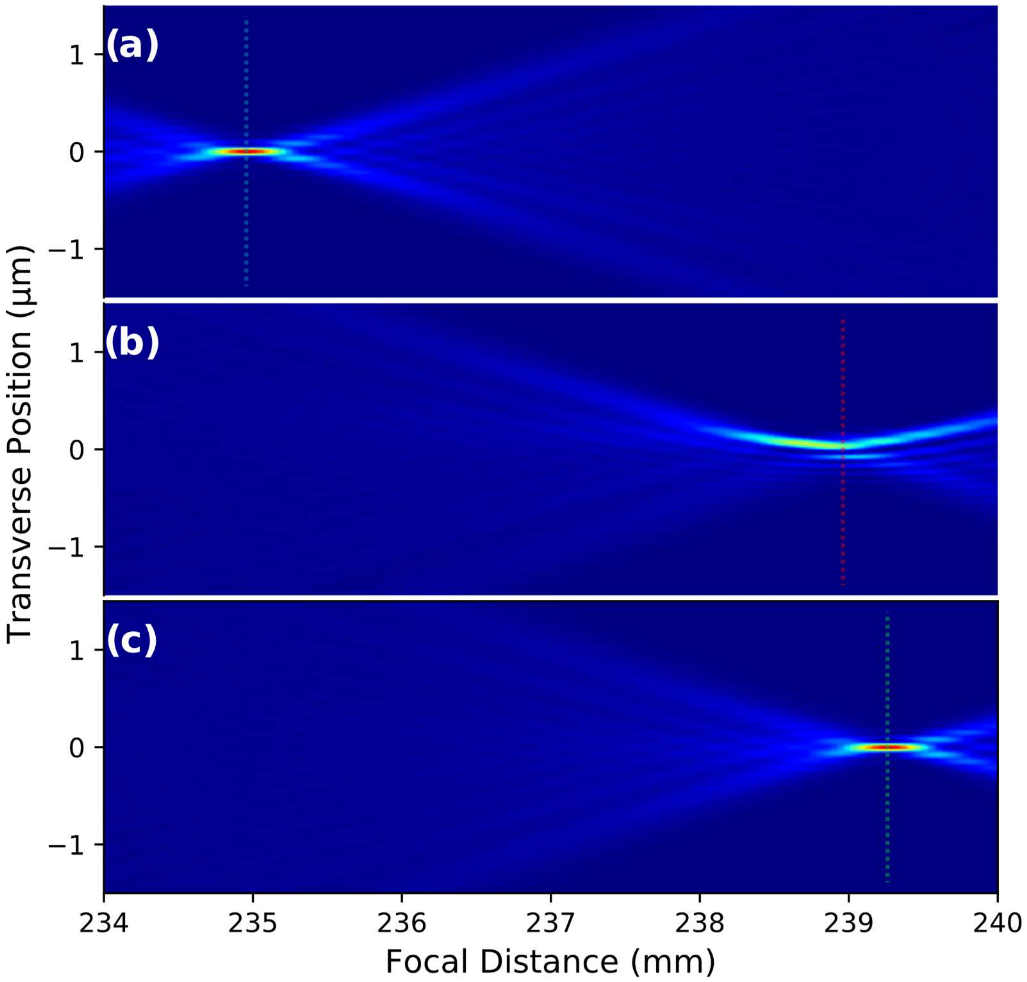

Supplementary Fig. 1: VKB beam caustics. The beam Intensity distribution after numerical propagation of the measured complex field to the intended VKB focal positions of (a) 235 mm ideal focus (b) 239.0 mm (only defocus correction) (c) 239.4 mm (defocus and coma corrections).

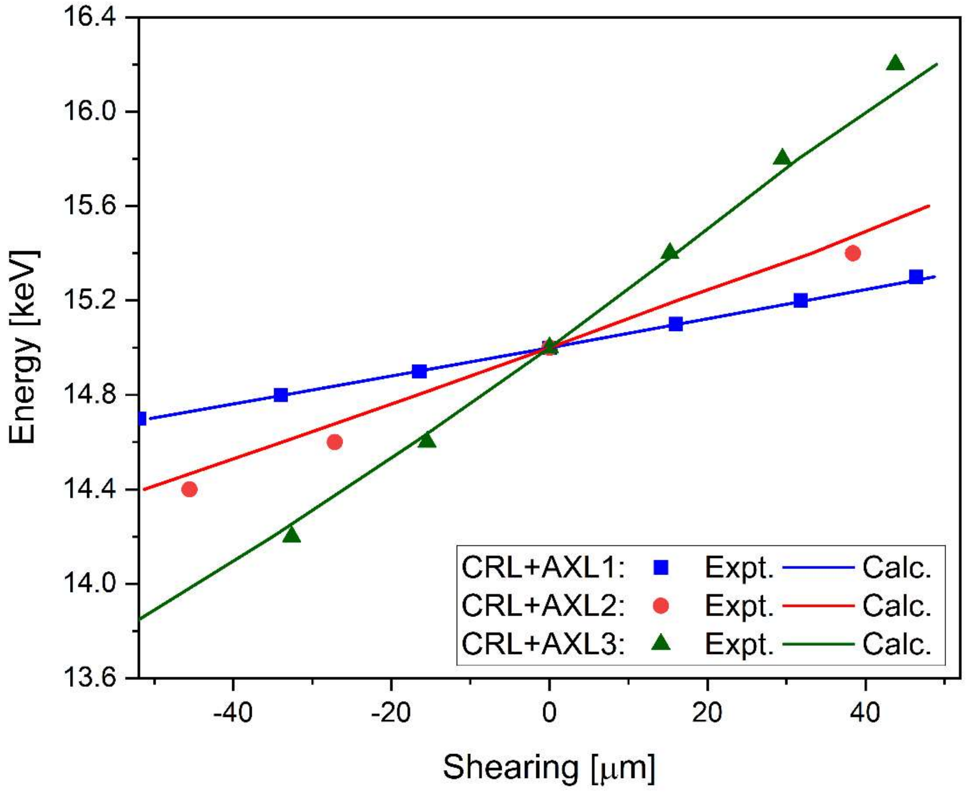

Supplementary Fig. 2: AXL calibration curve for different X-ray energies. The amount of AXL structures shearing required to compensate focal shift for discrete energy change.

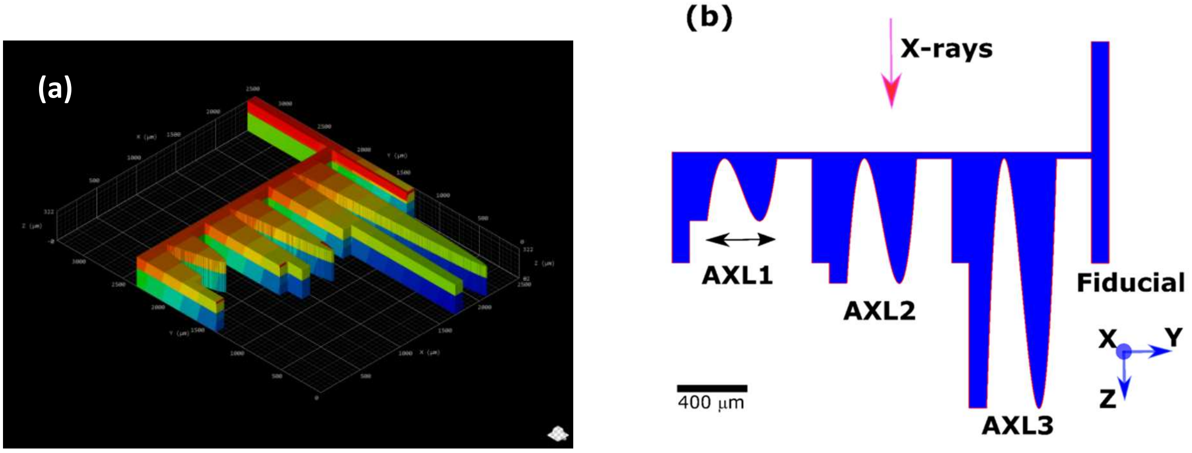

Supplementary Fig. 3: Design data of Structure-B of AXLs. Design layout of AXL1, AXL2, AXL3 in (a) 3-dimensions and (b) 2-dimensions. The geometric aperture of all AXLs was 400 μm. The fiducials shown above were used in achieving optimum alignment of the AXL structures.
